# A novel antibody–drug conjugate targeting SAIL for the treatment of hematologic malignancies

**DOI:** 10.1038/bcj.2015.39

**Published:** 2015-05-29

**Authors:** S Y Kim, J-W Theunissen, J Balibalos, S Liao-Chan, M C Babcock, T Wong, B Cairns, D Gonzalez, E H van der Horst, M Perez, Z Levashova, L Chinn, J A D‘Alessio, M Flory, A Bermudez, D Y Jackson, E Ha, J Monteon, M F Bruhns, G Chen, T-S Migone

**Affiliations:** 1 Department of Biology, Igenica Biotherapeutics, Burlingame, CA, USA; 2 Department of Preclinical Development, Igenica Biotherapeutics, Burlingame, CA, USA; 3 Department of Chemistry, Igenica Biotherapeutics, Burlingame, CA, USA; 4 Department of Process Development, Igenica Biotherapeutics, Burlingame, CA, USA

## Abstract

Although several new therapeutic approaches have improved outcomes in the treatment of hematologic malignancies, unmet need persists in acute myeloid leukemia (AML), multiple myeloma (MM) and non-Hodgkin's lymphoma. Here we describe the proteomic identification of a novel cancer target, SAIL (Surface Antigen In Leukemia), whose expression is observed in AML, MM, chronic lymphocytic leukemia (CLL), diffuse large B-cell lymphoma (DLBCL) and follicular lymphoma (FL). While SAIL is widely expressed in CLL, AML, MM, DLBCL and FL patient samples, expression in cancer cell lines is mostly limited to cells of AML origin. We evaluated the antitumor activity of anti-SAIL monoclonal antibodies, 7-1C and 67-7A, conjugated to monomethyl auristatin F. Following internalization, anti-SAIL antibody–drug conjugates (ADCs) exhibited subnanomolar IC_50_ values against AML cell lines *in vitro*. In pharmacology studies employing AML cell line xenografts, anti-SAIL ADCs resulted in significant tumor growth inhibition. The restricted expression profile of this target in normal tissues, the high prevalence in different types of hematologic cancers and the observed preclinical activity support the clinical development of SAIL-targeted ADCs.

## Introduction

In recent years, the development of antibody–drug conjugates (ADCs) has become an effective approach for the treatment of cancer.^1, 2, 3, 4^ The ability to combine the specificity of an antibody directed to a cell surface antigen with the cytotoxicity of potent small molecular weight drugs, such as tubulin inhibitors and DNA cross-linking agents, has been demonstrated to confer an improved therapeutic index compared with more traditional chemotherapeutic agents.^1, 2, 3, 4^ The regulatory approvals of brentuximab vedotin and ado-trastuzumab emtansine have demonstrated that ADCs can provide significant clinical advantages compared with unconjugated antibodies.^4, 5, 6^ There are currently more than 35 ADCs in clinical development,^7, 8^ and even though some promising results have been reported, the available data suggest that developing highly efficacious therapeutics through this modality may be more complex than initially expected.^9^

One of the main challenges in the development of novel ADCs is the identification of a cell surface protein that is selectively expressed in tumors and that allows for efficient internalization of the payload to provide a clinical benefit.^10^ Another challenge is to couple a highly specific monoclonal antibody (mAb) to the appropriate linker–toxin combination to achieve the desired safety and efficacy profile.^11^

Here we describe the proteomic identification of the novel cell surface antigen SAIL (Surface Antigen In Leukemia) and the preclinical characterization of ADCs with potent *in vitro* and *in vivo* activity against SAIL-expressing hematologic tumors.

## Materials and methods

### Cell lines

All human cell lines were purchased from the American Type Culture Collection (Manassas, VA, USA), Deutsche Sammlung von Mikroorganismen und Zellkulturen (Braunschweig, Germany) or the Japanese Collection of Research Bioresources Cell Bank (JCRB; Osaka, Japan) and were maintained as recommended.

### Patient samples and normal controls

Procedures to obtain specimens were conducted under institutional review board approval with all patients signing informed consent. Fresh specimens from acute myeloid leukemia (AML) and multiple myeloma (MM) patients and normal peripheral blood mononuclear cells (PBMCs) and bone marrow mononuclear cells (BMMCs) from nondiseased donors were acquired from AllCells (Emeryville, CA, USA). Fresh chronic lymphocytic leukemia (CLL) specimens were from Billings Clinic (Billings, MT, USA) and the University of Florida. Additional frozen AML and CLL patient specimens for flow analysis were from AllCells and the University of California San Diego, respectively. Primary solid tumors and normal adjacent control samples were from CHTN (The Cooperative Human Tissue Network) or the National Disease Research Interchange. CHTN is funded by the National Cancer Institute.

### Surface-tagged antigen analysis and liquid chromatography-coupled tandem mass spectrometry

Specimens were received within 6–24 h of sample collection. Upon receipt, the specimens were surface labeled using methods similar to those previously described.^12^ Before labeling, solid tumor specimens and adjacent tissues were mechanically and enzymatically dissociated and the samples were chromatographically enriched for tagged proteins using a solid-phase affinity resin.

Eluted proteins from surface-tagged antigen were identified and quantitated using an LTQ-Orbitrap Velos Pro hybrid mass spectrometer (Thermo Fisher Scientific, Waltham, MA, USA) configured with an EASY-nLC (Thermo Fisher Scientific) instrument for in-line nanoflow liquid chromatography. Resulting data were searched against the Uniprot human FASTA database using the SEQUEST algorithm executed on the Sorcerer 2 platform (SageN Research, San Jose, CA, USA). The relative quantitative levels of identified proteins were determined using the spectral counting method.^13^ Spectral counts were tabulated and transformed to Percent Normalized Spectral Abundance Factor (% NSAF) values to account for differences in protein length and variability in sample input^14, 15^ using Scaffold software (Proteome Software, Portland, OR, USA). Statistical significance between groups was calculated using the Wilcoxon rank-sum test.

### Antibody generation and binding assays

SAIL-binding mouse mAbs were generated by standard hybridoma methodology after immunization with mouse sarcoma cells stably transfected with the human SAIL antigen.

Apparent antigen-binding Kd of anti-SAIL mAb 67-7A and 7-1C was established by peptide enzyme-linked immunosorbent assay (ELISA) or by cell-based flow cytometry methods.^16, 17^ For ELISA Kd studies, plates coated with human extracellular domain (ECD) peptide were incubated with increasing concentrations of antibodies. After incubation with an HRP-conjugated secondary antibody (Jackson Immunoresearch, WestGrove, PA, USA), luminescence data were obtained and used to calculate an apparent Kd with 95% confidence intervals using Prism software version 6 (GraphPad, San Diego, CA, USA). For the Kd studies using flow cytometry, mouse sarcoma cell lines engineered to express full-length human SAIL were incubated with increasing concentrations of antibodies. Cells were then incubated with an Alexa Fluor 647-conjugated secondary F(ab')2 specific for the mouse IgG Fc and flow cytometry analysis was conducted to calculate an apparent Kd using Prism software.

### Flow cytometric analysis and internalization assay

All cell lines and primary samples were stained at a saturating concentration of 10 μg/ml for 30 min on ice using Alexa Fluor 647-conjugated 7-1C antibody. Primary samples were co-stained with multiple tumor markers as described in the figure legend. Antibodies were purchased as follows, Miltenyi Biotec (Bergisch Gladbach, Germany): CD33 (catalog number 130-091-732), CD34 (130–095–393), CD38 (130-099-151); Biolegend (San Diego, CA, USA): CD5 (300622), CD19 (302208), CD56 (318332); eBioscience (San Diego, CA, USA): CD3 (48-0037-42), CD14 (25-0149), Lineage cocktail (22-7778-72). 7-1C and isotype matched control-Alexa Fluor 647 were prepared using the Alexa Fluor 647 Antibody Labeling Kit (Life Technologies, Carlsbad, CA, USA). SAIL copy number was determined by interpolation on a calibration curve generated by Quantum Simply Cellular bead standards (Bangs Laboratories Inc., Fischers, IN, USA). Data acquisition was performed using a MACS-Quant 10 (Miltenyi Biotec) and all data analysis was performed using FlowJo 9.4.11 (Flowjo LLC, Ashland, OR, USA).

Antibody internalization studies were performed as previously described.^18^ In brief, the cells were incubated with 7-1C-Alexa Fluor 488 antibody at a saturating concentration of 15 μg/ml on ice for 1 h, washed and incubated at 37 °C for the indicated times. Following incubation, cells were immediately chilled and surface quenched for 30 min on ice using anti-Alexa Fluor 488 Rabbit IgG (30 μg/ml, Life Technologies, catalog number A11094) and analyzed by flow cytometry.

### RNA *in situ* hybridization

All formalin-fixed, paraffin-embedded tissue microarrays for lymphoma and normal tissues were obtained from TriStar Technology Group (Rockville, MD, USA) and US Biomax (Rockville, MD, USA). *In situ* SAIL RNA analysis on microarrays was performed using the RNAscope technology as previously described (Advanced Cell Diagnostics, Hayward, CA, USA).^19^ RNA ISH (*in situ* hybridization) was typically performed in parallel with positive control peptidylpropyl isomerase B to assess tissue RNA integrity. Staining intensity for ISH was scored as 0 (negative), 1 (weak), 2 (moderate) or 3 (strong). Images were acquired at a digital magnification of × 120 using Nanozoomer slide scanner software (Hamamatsu Photonics, Hamamatsu, Japan).

### Generation of ADCs

ADCs of anti-SAIL mouse mAbs (67-7A and 7-1C) were generated by conjugating an average of 3.5 monomethyl auristatin F (MMAF) molecules per antibody. MMAF was conjugated to the cysteine residues via a maleimidocaproyl (mc) linker as previously described.^20^

### Cytotoxicity assays with hematologic malignant cell lines and normal PBMC subsets

To assess ADC cytotoxicity, cells were plated in 384-well plates (Greiner Bio-One, Monroe, NC, USA) at 4000 cells per well in 40 μl of media. MMAF-conjugated anti-SAIL antibodies were serially diluted from 250 nM (except for experiments on sarcoma cells which started at 900 nM) and added to appropriate wells in duplicate. Cell plates were then incubated for 3 days, followed by lysis in CellTiter-Glo (CTG) assay reagent (Promega, Madison, WI, USA). CTG luminescence was quantified on a Synergy HT plate reader (BioTek, Winooski, VT, USA) and graphed. IC_50_ and s.e.m. were calculated using Prism's nonlinear curve fitting.

Monocytes and T cells were isolated from PBMCs by using the Pan Monocyte and Pan T-cell Isolation Kits (Miltenyi Biotec). Monocytes (7500) and 1000 T cells were plated in RPMI-1640 with Glutamax (Life Technologies), 10% heat-inactivated human AB serum (MP Biomedicals, Burlingame, CA, USA) and 100 μg/ml primocin (Invivogen, San Diego, CA, USA) in 384-well plates. T-cell proliferation was induced with CD2/CD3/CD28 activation beads and 20 ng/ml IL-2 (Miltenyi Biotec) and the monocyte phenotype was maintained with 10 ng/ml M-CSF (Peprotech, Rocky Hill, NJ, USA). After a 3-day incubation with 67 nM of ADC, 25 nM free MMAE, or 4.1 μM cytarabine, viability was assessed with CTG assay reagent. The CTG luminescence data was normalized against a no-treatment control. One representative experiment of multiple is shown.

OCI-AML3 cells stably transduced with MISSION lentiviral particles expressing short hairpin RNA targeting SAIL were generated according to manufacturer's instructions (Sigma-Aldrich, St Louis, MO, USA). Stably transfected populations were selected in 0.2 μg/ml puromycin for 7 days before the cytotoxicity assay. One representative experiment of two is shown.

### *In vivo* studies

Six- to 8-week-old female CB17 severe combined immunodeficiency mice were obtained from Charles River (Wilmington, MA, USA). Subcutaneous tumors were generated by an injection of 1 × 10^7^ cancer cells/mouse in a mixture of phosphate-buffered saline (without magnesium or calcium) and BD Matrigel (BD Biosciences, San Jose, CA, USA) at a 1:1 ratio in the right flank. Mice were randomized when tumors reached a size of 65–200 mm^3^ into treatment groups. The animal studies contained the following number of mice per experimental point: Figure 6a, *n*=9; Figure 6b, *n*=9; Figure 6c, *n*=6. ADCs 67-7A-mcMMAF, 7-1C-mcMMAF and isotype-mcMMAF (3 mg/kg) and cyclophosphamide (150 mg/kg) were administered intravenously (2 weekly doses). Body weights and tumors were measured in a nonblinded manner once and twice weekly, respectively. Tumor volume was calculated as described.^21^ Statistical significance between treatment and control groups was calculated using the Student's two-tailed *t*-test (Prism software). A *P*-value <0.05 was considered statistically significant. The animal experiments in this publication were performed in accordance with protocols approved by the Igenica Biotherapeutics Institutional Review Board - Animal Care and Use Committee.

## Results

### Proteomic identification of the novel surface antigen SAIL

The surface-tagged antigen procedure and high-resolution mass spectrometry were used to identify cell surface targets expressed on tumor cells, an approach that offers an important advantage over RNA-based procedures in that cell surface proteins are directly interrogated for expression.^22, 23, 24^ The method, adapted for interrogation of primary tissue specimens, employed freshly isolated primary tumors and normal tissues as source material for cell surface protein profiling. Biotinylation of intact cells from patients and nondiseased donors, followed by isolation of labeled proteins, allowed for the enrichment of cell surface proteins before the analysis by mass spectrometry.

**Figure 1:**
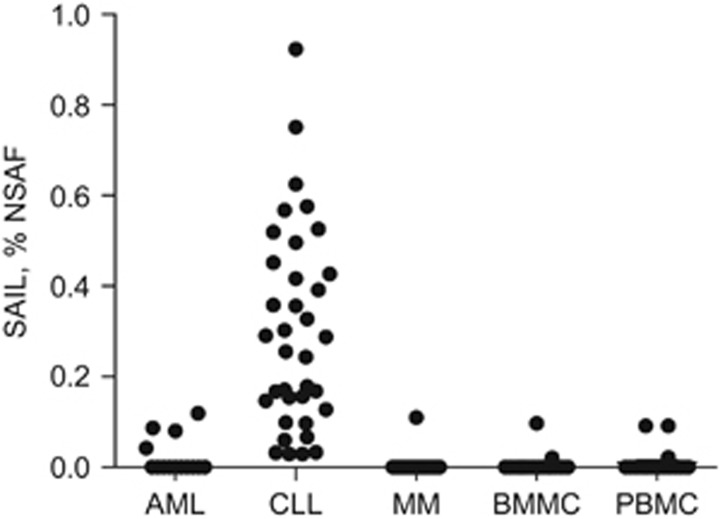
Proteomic identification of SAIL in hematologic malignancies. Expression of SAIL was analyzed in 14 AML, 40 CLL and 33  MM patient specimens, as well as in 21 normal BMMC and 20 normal PBMC controls. The relative quantitative protein abundance was determined using mass spectrometry-based spectral counting. Raw spectral counts were calculated as % NSAF.

A novel protein (C16orf54, Uniprot Accession Number Q6UWD8), hereafter referred to as SAIL, was first identified based on its high expression in CLL primary samples (Figure 1, Supplementary Table 1). Normalized spectral counts (converted to % NSAF) were significantly elevated in 36 out of 40 CLL samples compared with normal PBMC and BMMC controls. SAIL was also detected in primary AML samples (4 out of 14) and MM samples (1 out of 33). The proteomic evaluation was expanded to various types of solid tumors and normal adjacent tissues, including colorectal, lung, ovarian, pancreatic and sarcoma cancer samples, but SAIL was not detected in either these malignant or normal tissues within the limits of assay sensitivity^25, 26^ (Supplementary Table 1). The restricted expression profile of this novel cell surface antigen in normal tissue and its abundance in CLL provided the rationale for investigating its potential as an antibody target.

SAIL is a 224 amino-acid (aa) protein with one predicted transmembrane domain and a 31 aa ECD (Supplementary Figure 1). Before our proteomic identification of SAIL in hematologic cancer samples, it had previously been described as a transcriptional target of RUNX1/AML1 expressed during the development of the mouse hematologic system.^27^

### Generation and characterization of antibodies against SAIL

A panel of 277 mouse antibodies against SAIL was generated by immunizing mice with syngeneic cells stably expressing full-length human SAIL. Antibodies 7-1C and 67-7A were selected as lead therapeutic candidates based on binding properties during screening. More detailed binding studies of the two lead mAbs were conducted in ELISA assays that employed a peptide corresponding to the ECD region of SAIL (Table 1). Both 7-1C and 67-7A bound the ECD peptide with apparent Kd values ranging between 1.2 nM and 1.6 nM. Binding characteristics were further evaluated in flow cytometry analyses performed with cells stably expressing SAIL. The Kd values for the full-length SAIL antigen were comparable to those measured with the ECD peptide (Table 1).

### SAIL expression in hematologic malignancies and in normal tissues

**Figure 2:**
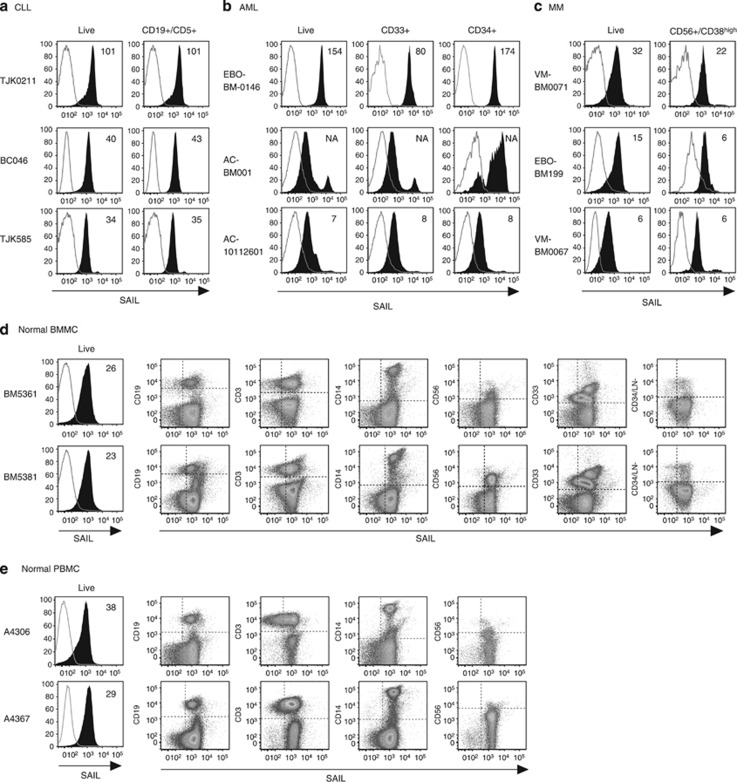
Cell surface expression of SAIL in CLL, AML and MM patient samples and normal BMMC and PBMC controls. (**a**) Three CLL specimens analyzed by flow cytometry. CLL cells were identified as CD19/CD5 double-positive cells. The histograms present SAIL (filled) and isotype control (open) staining in the live-cell and the CLL population. (**b**) Flow cytometry analysis of three AML specimens. SAIL expression is assessed in live-cells, CD33-positive and CD34-positive cells. (**c**) Flow cytometry analysis of three  MM specimens. CD38^high^ cells with CD56 expression were gated for MM cells. SAIL expression is assessed in the live-cell and the MM population. (**d** and **e**) Flow cytometry analysis of SAIL expression in BMMC (**d**) and PBMC (**e**) via co-staining with CD19, CD3, CD14, CD56, CD33, CD34 and a cocktail of lineage (LN) markers. Numbers in histograms are median-fluorescence-intensity fold-change values relative to the isotype control. Three and two representative examples are shown for the tumor and normal samples, respectively.

To corroborate the MS data, SAIL expression in primary AML, CLL and MM tumor samples was evaluated by flow cytometry. Uniform cell surface expression of SAIL was observed in all the CLL samples (*n*=20) evaluated by flow cytometry (Figure 2a), whereas more variable expression was noted in AML (*n*=13) and MM (*n*=7) primary samples (Figures 2b and c). These findings were consistent with what was observed during the proteomic analysis and may reflect the more heterogeneous phenotype of AML and MM patient populations, compared with the CLL patient population. SAIL expression was detected in multiple hematologic cell subpopulations in normal PBMC (*n*=6) and BMMC (*n*=5) specimens by flow cytometry analysis (Figures 2d and e). Within the limits of assay sensitivity, mass spectrometry detected SAIL expression in at least 10% of normal PBMC and BMMC controls (Supplementary Table 1).^25, 26^

**Figure 3:**
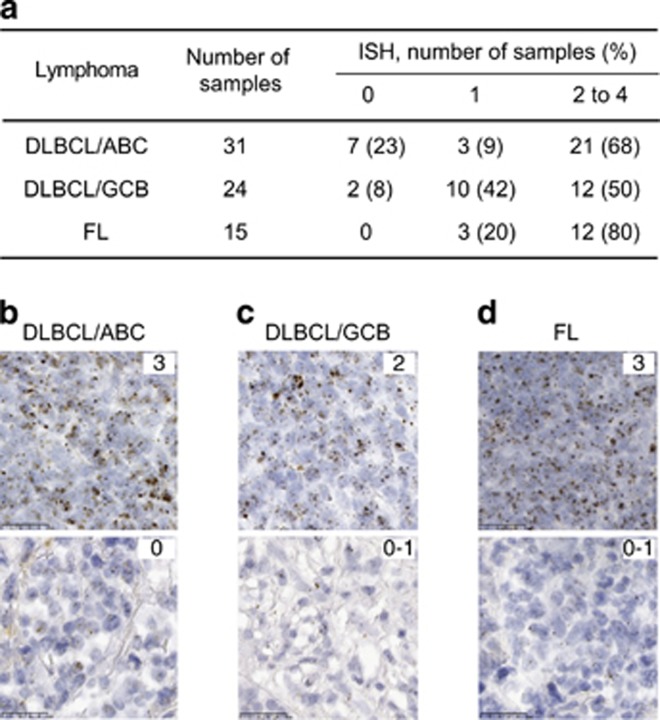
RNA ISH analysis of SAIL expression in non-Hodgkin's lymphoma tissue microarrays. (**a**) The number of samples tested for each tumor type is shown along with the percentage of ISH scores on a 0 to 3 scale. Examples of strong (score 3) and negative/weak (score 0–1) SAIL expression in the ABC (**b**) and GCB (**c**) subtypes of DLBCL and FL (**d**).

To assess whether SAIL was present in hematologic malignancies beyond the initial three types evaluated, an expanded analysis of lymphoma samples was performed by RNA ISH analysis (Figure 3). Eighty-seven percent prevalence of positive SAIL expression was noted in B-cell lymphomas by ISH analysis. Of particular interest was the fact that in addition to the follicular lymphoma (FL) samples, the majority of samples of both the activated B-cell (ABC) and germinal center B-cell (GCB) subtypes of diffuse large B-cell lymphoma (DLBCL) exhibited SAIL staining.

To confirm the proteomic data that suggested SAIL expression is minimal in normal tissues, an evaluation of normal tissue microarrays was performed using ISH (Supplementary Table 2). SAIL expression was noted in lymphoid tissues (lymph node, spleen, thymus and tonsil), whereas epithelial staining was sparse in five tissues (cervix, esophagus, gallbladder, pancreas and uterus) and high in one tissue (bladder urothelium). SAIL mRNA expression was undetectable by ISH in 13 other normal epithelial tissues.

### *In vitro* evaluation of anti-SAIL ADCs

**Figure 4:**
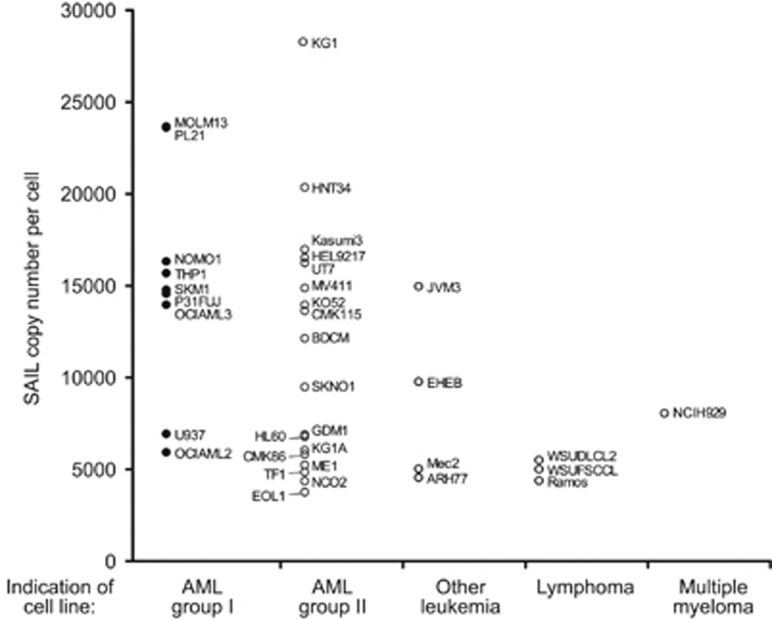
Quantitative flow cytometry analysis of SAIL expression in hematologic tumor cell lines. SAIL expression was analyzed in anti-SAIL ADC-sensitive (AML group I, black circle) and -nonsensitive (AML group II, other leukemia, lymphoma and MM, empty circle) cells. Cell lines in which the specific ADC IC_50_ was achieved at concentrations greater than 1 nM were considered nonsensitive. Data represent the mean of at least two independent fluorescence quantitation experiments per cell line. Only the cell lines with greater than 3000 copies per cell were considered for this analysis.

In contrast to the high prevalence of expression observed in primary B-cell lymphoma samples, SAIL expression in cancer cell lines was found to be mostly restricted to lines of the myeloid lineage. SAIL copy-number enumeration by flow cytometry showed that the antigen is moderately expressed on most cell lines with copy numbers ranging between 5000 and 30 000 (Figure 4).

**Figure 5:**
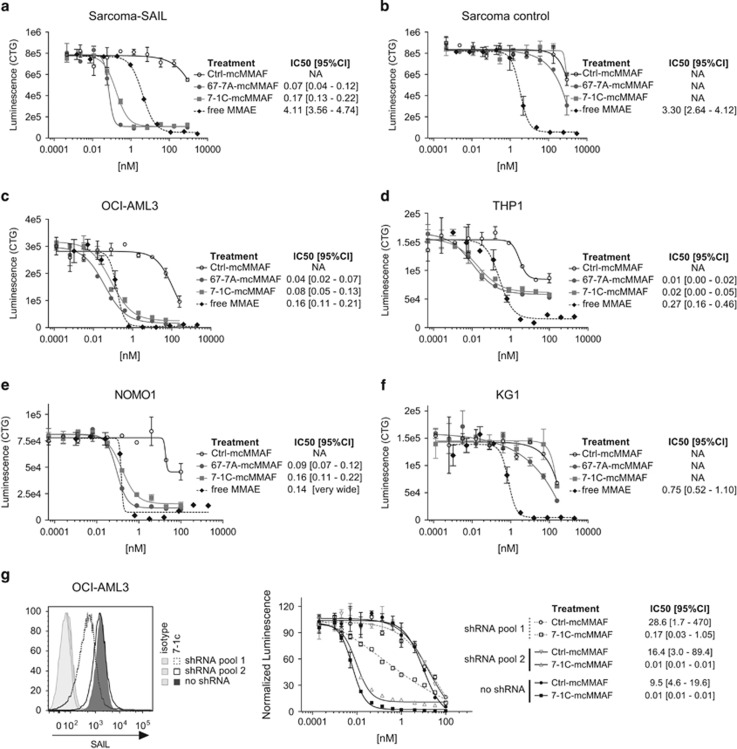
*In vitro* activity of 67-7A-mcMMAF and 7-1C-mcMMAF in tumor cell lines. Upon incubation of cells for 96 h with serial dilutions of 67-7A-mcMMAF (gray circle), 7-1C-mcMMAF (gray square), a nonspecific (ctrl) mouse IgG2a-mcMMAF (empty circle) or free MMAE (black diamond), cell viability was assessed by measurement of the ATP levels (Luminescence (CTG)). Mouse sarcoma cells transfected with SAIL (**a**), untransfected mouse sarcoma cells (**b**), OCI-AML3 (**c** and **g**), THP1 (**d**), NOMO1 (**e**) and KG1 (**f**). (**g**) Fluorescence intensity of SAIL in OCI-AML3 cells that were transduced by short hairpin RNAs (shRNAs) targeting SAIL compared with no shRNA transduced cells (left). *In vitro* activity of 7-1C-mcMMAF for OCI-AML3 cells with (shRNA pool 1) or without (shRNA pool 2 or no shRNA) SAIL knockdown (right). Results are the mean±s.e.m. of duplicate samples. The IC_50_ values and their associated 95% confidence intervals (95% CIs) are reported. NA, not applicable.

Anti-SAIL ADCs were generated by conjugating mcMMAF to mAbs 7-1C and 67-7A. These ADCs were evaluated for cytotoxicity against sarcoma cells overexpressing the target and against cell lines that had undergone copy-number analysis. The SAIL-specific ADCs exhibited potent *in vitro* cytotoxic activity against the sarcoma cell line expressing high levels of SAIL, but had no effect on the nontransfected parental sarcoma line, demonstrating target-specific cytotoxicity (Figures 5a and b). Across a panel of cancer cell lines, copy number generally did not correlate with ADC activity *in vitro*, a phenomenon observed for ADCs against other targets.^16, 28^ For example, KG1 cells were found to express the highest levels of SAIL yet were resistant to cell killing by the ADC, despite their sensitivity to free (unconjugated) monomethyl auristatin E (MMAE) (Figures 4 and 5f). Conversely, the ADCs demonstrated potent cytotoxic activity against the monocytic NOMO1, THP1 and OCI-AML3 cell lines, which express lower amounts of SAIL (14000 to 16000 copies per cell) compared with KG1 (Figures 5c, 5d and 5e versus 5f).

Because ADCs generally rely on internalization for cytotoxicity, one sensitive and one nonsensitive AML cell line were evaluated for anti-SAIL mAb internalization. Flow cytometry-based internalization assays showed that anti-SAIL mAb 7-1C was internalized by OCI-AML3 but not by KG1 cells, supporting the hypothesis that sensitivity to anti-SAIL ADCs is driven by internalization (Supplementary Figure 2).

Impact of the anti-SAIL ADC on normal hematologic cell populations with the highest degree of antigen expression, including monocytes and proliferating T cells, was evaluated *in vitro*. These PBMC subpopulations were not found to be sensitive to anti-SAIL ADC (Supplementary Figure 3). Whereas free MMAE affected viability by at least 50%, the anti-SAIL ADC did not affect viability at a higher molar concentration, indicating that the anti-SAIL ADC showed selectivity toward a subset of AML models.

To confirm that the activity observed against the AML cell lines was dependent on SAIL expression, knockdown experiments utilizing SAIL-specific short hairpin RNA constructs were performed in OCI-AML3 cells. In these studies, a 3.5-fold reduction in SAIL protein expression by flow cytometry was achieved and correlated with decreased anti-SAIL ADC activity (Figure 5g, short hairpin RNA pool 1).

Taken together, these data demonstrated that anti-SAIL ADCs were antigen-specific and exhibited potent cytotoxic activity toward AML cell lines *in vitro*.

### *In vivo* efficacy of anti-SAIL ADCs

**Figure 6:**
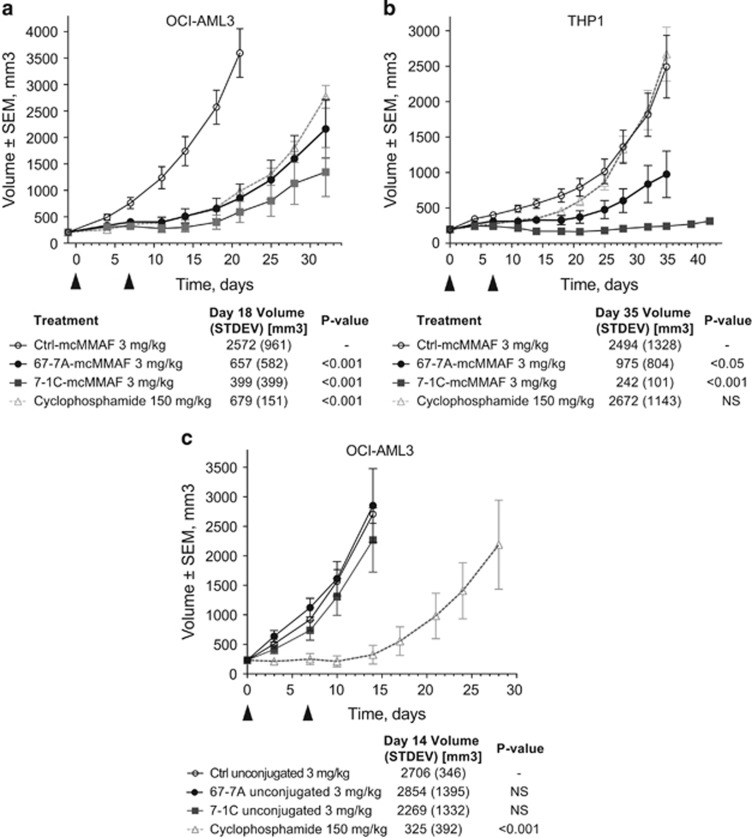
*In vivo* efficacy of 7-1C-mcMMAF and 67-7A-mcMMAF in AML xenograft models. Six- to 8-week-old CB17 mice were subcutaneously injected with OCI-AML3 (**a** and **c**) or THP1 (**b**) cells. (**a** and **b**) Four groups of six to nine mice were treated with 3 mg/kg of control-mcMMAF (empty circle), 67-7A-mcMMAF (black circle) and 7-1C-mcMMAF (gray square) or 150- mg/kg cyclophosphamide (empty triangle) according to the schedule indicated by the arrowheads. Data shown in **a** and **b** are from one representative of two independent experiments. (**c**) CB17 mice injected with OCI-AML3 were treated with 3 mg/kg of unconjugated control antibody (empty circle), 67-7A (black circle) and 7-1C (gray square) or 150 mg/kg cyclophosphamide (empty triangle) according to the schedule indicated by the arrowheads. No activity was detected with unconjugated antibodies compared with isotype control antibody. The tumor values at the end of study (mean and standard deviation (STDEV)) are used to calculate significance. NS, not significant.

To assess the antitumor activity of the anti-SAIL ADCs *in vivo*, two subcutaneous cell line xenograft models, chosen based on SAIL expression and ADC sensitivity *in vitro*, were utilized. In the OCI-AML3 and THP1 AML models, significant tumor growth inhibition was observed for the anti-SAIL ADCs compared with the nontargeting isotype control ADC at a dose level of 3 mg/kg (Figure 6a and b). In contrast, naked (that is, unconjugated) 67-7A and 7-1C did not exhibit significant activity in the OCI-AML3 model (Figure 6c), indicating that the main antitumor mechanism of the ADC is through the specific delivery of conjugated cytotoxic payload into tumor cells.

## Discussion

Antibody–drug conjugates represent a promising new class of anticancer therapeutics that combine the specificity of an antibody with the potent activity of cytotoxic drugs. Many of the ADCs that are in clinical development are based on antigens that had previously been tested as targets for traditional unarmed antibody therapeutics. For example, Her2 and CD30 are both cancer targets against which naked antibodies had been evaluated in a clinical setting before the development of the ADCs ado-trastuzumab emtansine and brentuximab vedotin, respectively.^29, 30, 31^ More recent research has focused on identifying targets whose biological role in cancer may be unknown, but whose expression profile is mostly restricted to malignant tissue.

A proteomics approach is ideally suited to identify membrane proteins that are differentially expressed in primary patient samples compared with samples from nondiseased donors.^22, 23, 24^ SAIL was first identified as part of a target discovery effort utilizing primary CLL samples, where it was found to be overexpressed in most tumors tested relative to normal PBMCs or BMMCs from nondiseased donors (Figure 1, Supplementary Table S1). While SAIL expression prevalence was 90% in CLL, only 29% of AML and 3% of MM specimens showed expression. It should be noted that the relatively low abundance of malignant cells in AML and MM samples may lead to an underestimation of protein expression levels of certain targets. Using this proteomics-based method, SAIL expression was not detected in a large set of colorectal, lung and ovarian cancer samples or their matched normal adjacent tissues. An analysis of SAIL expression in other solid tumor indications is currently in progress.

Antibodies against SAIL were developed using cell-based immunization methods in order to ensure that the protein would be recognized in the context of its native conformation at the cell surface. Two lead antibody clones, 67-7A and 7-1C, were selected for further characterization based on binding properties during the initial screening efforts. Binding affinity studies on cells and recombinant SAIL peptide demonstrated that both antibodies bound human SAIL with Kd values in the range of 0.6–1.6 nM.

Flow cytometry studies confirmed that SAIL was expressed in all CLL samples evaluated as well as in the majority of AML and MM patient samples (Figure 2). An expanded analysis of SAIL expression in hematologic malignancies by ISH showed that the transcript was expressed with very high prevalence (87%) in DLBCL and FL tumor specimens (Figure 3). The fact that SAIL was expressed at similar levels in both the ABC and GCB subtypes of DLBCL is of clinical interest, given the low success rate of R-CHOP shown to date in ABC-DLBCL.^32, 33^ The selective tumor expression of SAIL mRNA was also confirmed by an ISH analysis of normal tissues, which demonstrated expression predominantly in lymphoid tissues. Studies with immunohistochemical reagents are planned to confirm protein expression in DLBCL, FL and normal tissues.

An extensive analysis of SAIL expression in cancer cell lines was undertaken using a flow cytometry-based copy enumeration method. Interestingly, the SAIL expression pattern noted in primary lymphoma samples was not replicated in established lymphoma cancer cell lines, but was found to be mostly restricted to cancer cell lines of myeloid origin (Figure 4). In those cell lines, the copy number per cell was generally modest, ranging from 5000 to 30 000, similar to what has been reported for CD33, another clinical ADC target.^34^

The vast majority of ADCs currently in clinical development employ microtubule inhibitors (auristatins or maytansinoids) as cytotoxic payloads. For this reason, we decided to generate ADCs against SAIL by conjugating the two lead antibodies to the auristatin MMAF. The anti-SAIL ADCs showed cytotoxic activity against a subset of AML cell lines—OCI-AML3, THP1 and NOMO1 (Figure 5).

To understand what other factors may influence ADC response, flow cytometry-based internalization studies were performed on selected cell lines. Results demonstrated that the degree of cellular uptake of ADC correlated with its cytotoxic activity, consistent with findings for other targets like CD22.^28, 35^

In two AML cell line xenograft models, anti-SAIL ADCs were found to exhibit strong antitumor activity compared with a nonspecific isotype control ADC or with naked anti-SAIL mAbs (Figure 6). For the clinical development of a SAIL-targeted ADC therapeutic, humanized antibody variants are being evaluated in order to minimize potential immunogenicity in humans. In addition, site-specific conjugation approaches have been demonstrated to achieve a superior therapeutic index compared with conventional conjugation approaches,^36^ and therapeutic anti-SAIL ADC candidates under development may harness similar site-specific ADC technology.

In conclusion, we have characterized the expression of a novel ADC target, SAIL. Expression of SAIL is restricted in normal tissues but is present in different types of hematologic malignancies. Moreover, we have demonstrated that ADCs against SAIL have high *in vitro* potency and demonstrate high antitumor activity *in vivo* in multiple xenograft models. Given the body of data generated and the unmet clinical need in indications where SAIL is expressed with high prevalence, clinical development of anti-SAIL ADCs may be warranted.

## Figures and Tables

**Table 1:** Affinity of 7-1C and 67-7A to human SAIL antigens

*mAb*	*ELISA Kd using ECD peptide, nM (95% confidence interval)*	*Flow Kd using Sarcoma-SAIL, nM (95% confidence interval)*
7-1C	1.21 (1.03–1.40)	0.57 (0.47–0.68)
67-7A	1.62 (1.17–2.06)	0.57 (0.45–0.68)

Abbreviations: ECD, extracellular domain; ELISA, enzyme-linked immunosorbent assay; mAb, monoclonal antibody; SAIL, Surface Antigen In Leukemia.

Data from one of multiple experiments are shown.

## Notes

All authors are employees of Igenica Biotherapeutics. Igenica Biotherapeutics is a company funded by venture capital firms – The Column Group, 5AM Ventures, OrbiMed and Third Rock Ventures.
