# Supplementary material for: A novel antibody–drug conjugate targeting SAIL for the treatment of hematologic malignancies
Source: Blood Cancer J. 2015 May 29;5(5):e316–. doi: 10.1038/bcj.2015.39 (PMC4476018; doi:10.1038/bcj.2015.39)
Supplement: Supplementary Figure S1 [file bcj201539x1.pdf]

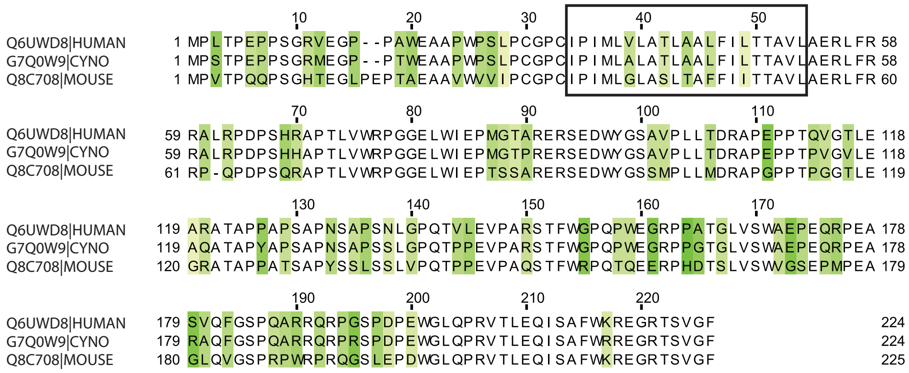

Figure S1. Multiple sequence alignment of SAIL protein between human, cynomologus monkey and mouse.
The Uniprot Accession Numbers for the input sequences are shown. Green-shaded boxes indicate divergent sequences.
Predicted transmembrane domain is highlighted by black box.
