# Supplementary material for: A novel antibody–drug conjugate targeting SAIL for the treatment of hematologic malignancies
Source: Blood Cancer J. 2015 May 29;5(5):e316–. doi: 10.1038/bcj.2015.39 (PMC4476018; doi:10.1038/bcj.2015.39)
Supplement: Supplementary Figure S2 [file bcj201539x2.pdf]

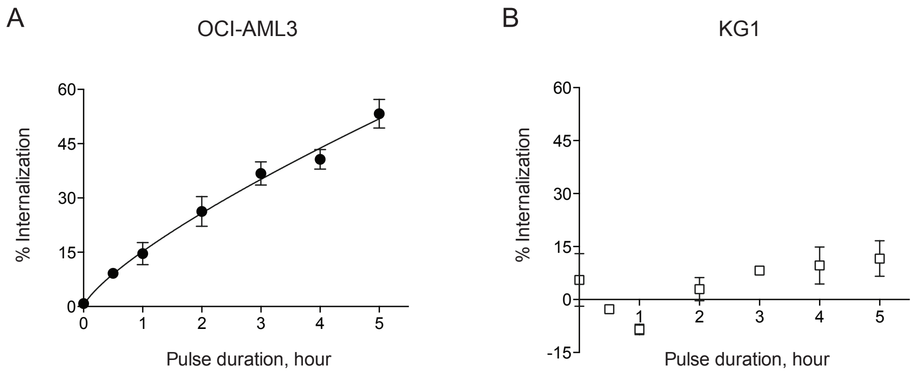

Figure S2. Internalization of 7-1C in OCI-AML3 (A) and KG1 (B). Cells were labeled with 7-1C-Alexa Fluor 488 on ice, incubated at 37°C for the indicated times and placed on ice with or without surface quenching by anti-Alexa Fluor 488 IgG. Median fluorescence intensity (MFI) from flow cytometry analysis was normalized to the initial surface binding as described in [18]. Results are the mean ± SEM of duplicate samples. The curve fitting of the data was conducted using Prism's nonlinear regression analysis.
