# Supplementary material for: A novel antibody–drug conjugate targeting SAIL for the treatment of hematologic malignancies
Source: Blood Cancer J. 2015 May 29;5(5):e316–. doi: 10.1038/bcj.2015.39 (PMC4476018; doi:10.1038/bcj.2015.39)
Supplement: Supplementary Figure S3 [file bcj201539x3.pdf]

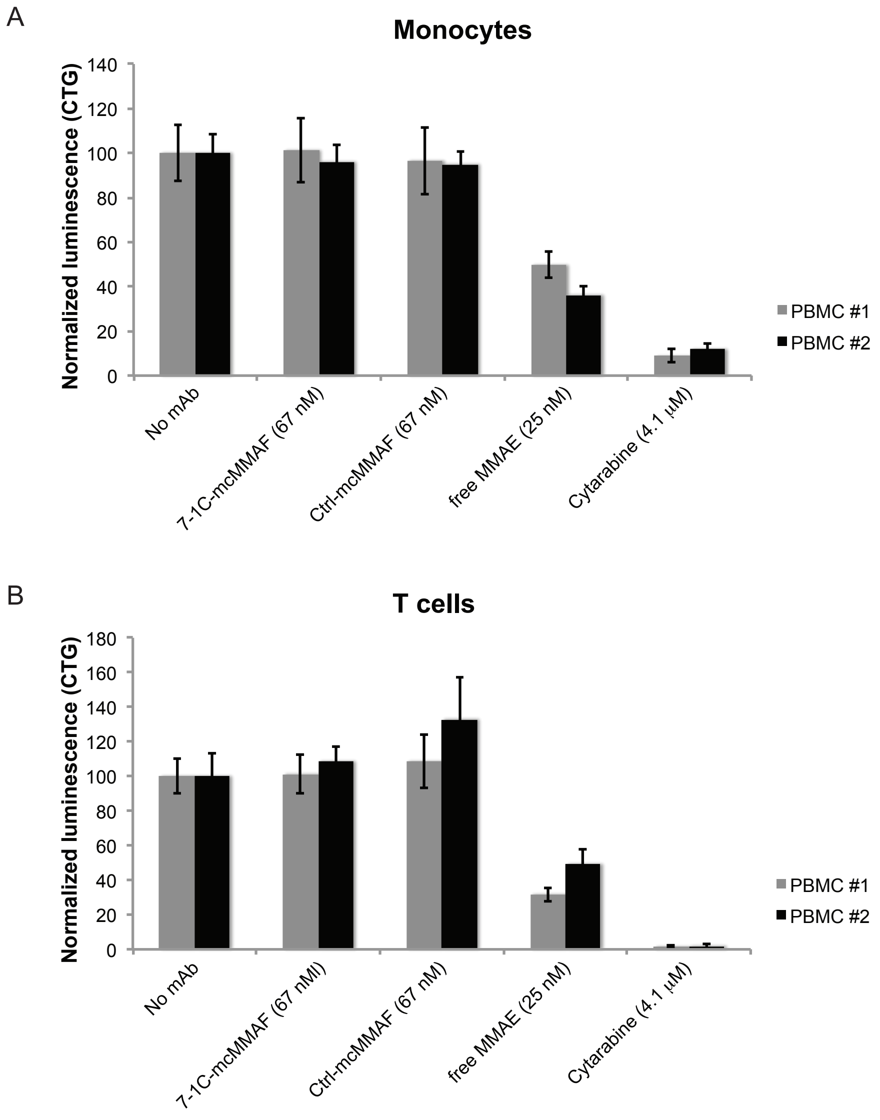

Figure S3. Anti-SAIL ADCs do not affect viability of monocytes and proliferating T cells.
Cell viability was assessed by measuring ATP levels in monocytes (A) or proliferating T cells (B) after 3 days of exposure to 7-1C-mcMMAF, a non-specific (ctrl) mouse IgG2a-mcMMAF, free MMAE or cytarabine. ATP levels [luminescence (CTG)] for each treatment were normalized against a no mAb treatment control. Results for two different PBMC donors are shown and the errors were estimated from the corresponding standard deviations.
