# Supplementary material for: A novel antibody–drug conjugate targeting SAIL for the treatment of hematologic malignancies
Source: Blood Cancer J. 2015 May 29;5(5):e316–. doi: 10.1038/bcj.2015.39 (PMC4476018; doi:10.1038/bcj.2015.39)
Supplement: Supplementary Table S1 [file bcj201539x4.pdf]

Cell/tissue type	Sample size (N)	SAIL			CD33		
		Mean %NSAF (STDEV)	P (two-sided)	Positive sample (%)	Mean %NSAF (STDEV)	P (two-sided)	Positive sample (%)
AML	14	0.02 (0.04)	NS	4 (28.6)	0.04 (0.05)	NS	6 (42.9)
Normal BMMC	21	0.01 (0.02)		2 (9.5)	0.01 (0.03)		6 (28.6)
CLL	40	0.27 (0.22)	<0.001	36 (90.0)	0	NA	1 (2.5)
Normal PBMC	20	0.01 (0.03)		3 (15)	0		4 (20.0)
MM	33	0.003 (0.02)	NS	1 (3.0)	0.06 (0.07)	<0.05	18 (54.5)
Normal BMMC	21	0.01 (0.02)		2 (9.5)	0.01 (0.03)		6 (28.6)
Colorectal tumor	27	0	NA	0	0	NA	0
Colorectal adjacent tissue	22	0		0	0		0
Lung tumor	89	0	NA	0	0	NA	0
Lung adjacent tissue	75	0		0	0		0
Ovarian tumor	53	0	NA	0	0	NA	0
Ovary adjacent tissue	10	0		0	0		0
Pancreatic tumor	41	0	NA	0	0	NA	0
Pancreas adjacent tissue	21	0		0	0		0
Sarcoma	14	0	ND	0	0	NA	0
Sarcoma adjacent tissue	ND	ND		ND	ND		ND

Table S1. Proteomic analysis of SAIL expression in various tumor and normal tissues. CD33 expression is shown as a comparator. The number of cases (N) tested for each tumor type is shown along with the mean of normalized spectral abundance factor (%NSAF), the standard deviation of %NSAF, the two-sided P-value (Wilcoxon rank sum test), and the percentage of positive samples. ND, not determined; NA, not applicable; NS, not significant.
