# Supplementary material for: A novel antibody–drug conjugate targeting SAIL for the treatment of hematologic malignancies
Source: Blood Cancer J. 2015 May 29;5(5):e316–. doi: 10.1038/bcj.2015.39 (PMC4476018; doi:10.1038/bcj.2015.39)
Supplement: Supplementary Table S2 [file bcj201539x5.pdf]

Tissue name	Sample size	Score
Adrenal gland	3	0
Bladder urothelium	3	1-3
Breast glands	1	0
Cervix epithelium	5	0-1
Colon mucosa	3	0
Esophageal epithelium	3	1
Gallbladder	3	1-2
Kidney cortex	3	0
Kidney medulla	2	0
Liver	1	0
Lung alveoli & epithelia*	2	0
Lymph node	3	2-3
Ovary	3	0
Pancreas acini & islets	3	1
Parotid gland	3	0
Prostate epithelia	2	0
Spleen	3	1
Stomach	3	0
Testis	3	0
Thymus	2	3
Thyroid	3	0
Tonsil	2	3
Uterus epithelia	3	0-1

Table S2. RNA ISH analysis of SAIL expression in normal tissues generated from specific tissue specimens and normal tissue TMAs (Tristar and US Biomax). Staining intensity was scored on a 0 to 3 scale as described in Material and Methods. * Macrophages ISH 2
